# Effect of a SILICA/HPAM Nanohybrid on Heavy Oil Recovery and Treatment: Experimental and Simulation Study

**DOI:** 10.1021/acsomega.4c03772

**Published:** 2024-08-30

**Authors:** Laura M. Corredor, Silvia Escobar, Janet Cifuentes, Sebastián Llanos, Henderson I. Quintero, Kelly Colmenares, Carlos Espinosa, Claudia L. Delgadillo, Arnold Rafael Romero Bohórquez, Eduardo Manrique

**Affiliations:** † Instituto Colombiano del Petróleo, ECOPETROL S.A., Piedecuesta 681011, Colombia; ‡ Universidad Industrial de Santander, Bucaramanga 680006, Colombia; § PSL proanálisis. Floridablanca 681001, Colombia; ∥ Meridian Consulting Ltd, Bogotá 110231, Colombia; ⊥ TIP LTDA, Girón, Bucaramanga 680002, Colombia; # Grupo de Investigación en Química Estructural, Departamento de Química, Universidad Industrial de Santander, Bucaramanga 680006, Colombia; ∇ Citation Oil & Gas Corporation, Houston 77069, United States

## Abstract

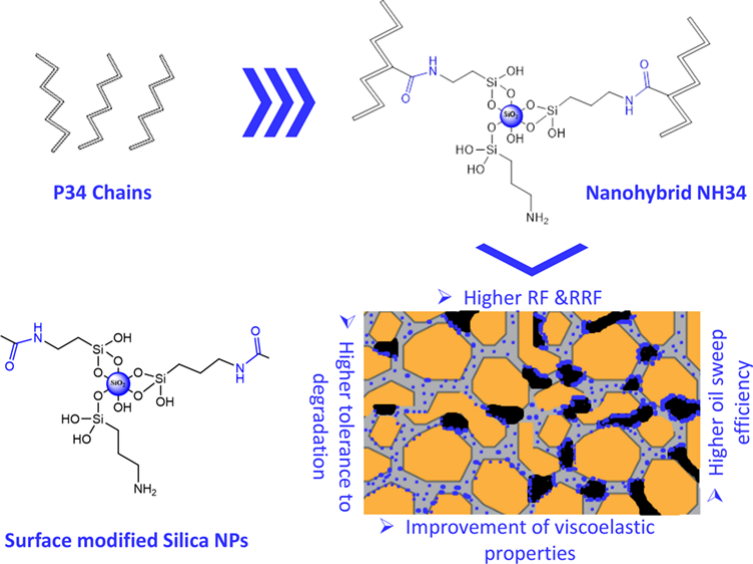

The addition of nanoparticles has been presented as an alternative approach to counteract the degradation of polymeric solutions for enhanced oil recovery. In this context, a nanohybrid (NH34) of partially hydrolyzed polyacrylamide (MW ∼12 MDa) and nanosilica modified with 2% 3-aminopropyltriethoxysilane (nSiO_2_-APTES) was synthesized and evaluated. NH34 was characterized by using dynamic light scattering, Fourier-transform infrared spectroscopy, and thermogravimetric analysis. Fluid-fluid tests assessed its viscosifying power, mechanical stability, filterability, and emulsion behavior. Rock-fluid tests were carried out to determine the nanohybrid's adsorption in porous media, the inaccessible pore volume (IPV), and the resistance (RF) and residual resistance factors (RRF). These tests were conducted under the conditions of a Colombian field. NH34 results were compared with four (4) commercial polymers (P34, P88, P51, and PA2). The viscosifying power of NH34 was observed to be similar to that of the four commercial polymers at a lower concentration, but it exhibits more resistance to mechanical and chemical degradation. The evaluation of the emulsion behavior showed that the nanohybrid neither changed the dehydration process nor altered the crude oil viscosity, favoring its extraction at the wellhead. However, the water clarification treatment must be adjusted because the oil and grease contents and turbidity increase with the residual concentration of NH34. Incremental oil recovery factors obtained by numerical simulation (compared to waterflooding) were P51 (5.5%) > P34 (4.9%) > P88 (4.8%) > NH34 (2.6%) > PA2 (0.9%). The polymers P51, P34, and P88 had a better recovery factor than NH34 and PA2 due to their lower values of residual adsorption and IPV. Few studies have been reported on polymer nanohybrids’ emulsion and flow behavior. Therefore, further research is needed to enhance our understanding of the fundamental enhanced oil recovery mechanisms associated with polymer nanohybrids.

## 1 Introduction

The effectiveness of polymer injection is constrained by the susceptibility of polyacrylamides, their copolymers, and terpolymers to mechanical, thermal, and chemical degradation, as indicated by a decrease in the viscosity of the polymeric solution. Incorporating nanoparticles (NPs) has emerged as a viable strategy to enhance the recovery efficiency of polymers in EOR processes. This enhancement occurs through several mechanisms, including optimizing the polymer solution’s rheological properties, mitigating polymer retention within porous media, altering wettability, and reducing interfacial tension.

Maghzi et al.^1^ and Bashir et al.^2^ reported that including SiO_2_, Al_2_O_3_, and TiO_2_ NPs enhances polyacrylamide solutions’ temperature and salinity resistance. This improvement arises from the NPs’ ability to hinder ion-dipole interactions between cations and the amide groups within the polymer, preventing the shielding effect and the loss of viscosity of the polymeric solution. This is because, in the presence of these NPs, ion-dipole interactions occur between cations and oxygen atoms on the surface of the NPs. Some studies have applied core–shell structures on the nanoscale to protect/transport the hydrophilic^3^ and hydrophobic^4^ polymer chains in harsh conditions (high temperature and high salinity, HTHS). Pu et al.^5^ reported a core–shell hyperbranched polymer (HBPAM) composed of a core surrounded by an outermost flexible shell. It was synthesized through free water radical polymerization to improve shear, salt, and temperature tolerance by incorporation of hydrophobic groups and thermosensitive pendants into the hydrophilic backbone of the polymer with 98% water cut reductions and oil efficiencies until 77% at 1750 ppm and 37.9 cP (0.25 PV). Liu et al.^6^ developed a core–shell structure, where a nanosilica is the central core, surrounded by a layer of amphiphilic polymeric chains.

The synthesis method involves a facile water-free radical polymerization process. This tailored material was specifically designed for applications in hostile reservoirs HTHS with oil recovery increases of 20% in sandstone cores at 1500 ppm and 57.6 cP (0.3 PV).

NPs also improve the rheological properties of the polymer by forming three-dimensional NP-polymer networks, where NPs act as cross-linkers between polymer chains.^7−9^ The structure of these networks is controlled by the size of the NP and the polymer, the amount of polymer adsorbed on the surface of the NP, the thickness of the adsorbed polymer, and the range of repulsion between particles.^10^ Rock-fluid evaluations have shown that electrostatic and steric forces on the surface of NPs caused by the presence of polymers provide a barrier to the adsorption of the nanohybrid in the porous media.^11−16^ Additionally, NPs can adsorb at the oil–water interface to reduce interfacial tension.^17−20^ The ability of NPs to decrease IFT can be enhanced through asymmetric surface modification or other surface modifications.^21^ The first method includes Janus-type NPs;^22−27^ the second contains NPs modified with polymers.^28−31^ NPs can also modify the wettability of the porous media due to their adsorption on the rock by electrostatic forces and improving the removal of crude oil and paraffins from the porous media.^28,32−36^

These mechanisms have been observed with nanofluids and nanohybrids.^36−38^ Nanofluids are dispersions of NPs in polymeric solutions, while in nanohybrids, NPs are bound to polymeric chains by covalent bonds.^39−41^ Before preparing the nanohybrid or nanofluid, the surface of the NPs is modified by chemical or physical methods to improve the compatibility between the two phases, with the chemical method being the most used because it inhibits the surface modifier’s desorption from the NPs. Nanohybrids based on polyacrylamide (PAM) have gained significant interest in enhanced oil recovery (EOR) due to their unique properties, high stability, and performance in harsh conditions^42^ and potential to improve oil recovery efficiency.^36,43^ Nanohybrids exhibit improved viscoelastic properties such as higher elasticity and shear thinning behavior, which are beneficial for better oil sweep efficiency;^44^ they can act as pore throat modifiers^45^ and O/W emulsion stabilizers,^46^ altering the flow characteristics and leading to improved polymer injectivity within the reservoir.^31,47^ Additionally, nanohybrids can act as highly efficient coagulants and flocculants due to their large surface area and tunable surface chemistry, allowing a quick agglomeration and settling,^43^ leading to improved clarification and sedimentation of suspended solids, organic matter, and other dispersed compounds in the water phase.^48^

Accordingly, the NH34 nanohybrid was synthesized to improve the performance of the P34 polymer for EOR applications. The nanohybrid’s viability as a recovery method was evaluated through viscosity tests, mechanical stability, filterability ratio, and emulsion behavior. The reservoir simulation shows that the injection of the nanohybrid and conventional polymers smooths the decline in the production curve and even reverses this decline. The performance difference between nanoparticle and polymer compounds was associated with the RRF and RF behavior.

## 2 Methodology

### 2.1 Materials

The nanohybrid was synthesized with NPs modified with 3-aminopropyltriethoxysilane (nSiO_2_-APTES NPs, 20 nm) supplied by NanoAmor (USA) and partially hydrolyzed polyacrylamide (P34) with a hydrolysis percentage between 25 and 35% and a molecular weight (MW) between 10 and 12 MDa. The reagents used were 2-propanol (C_3_H_8_O, >99.8%, Merck Millipore, USA), tetrahydrofuran (THF, 99.9%, Sigma-Aldrich, USA), and sulfuric acid (H_2_SO_4_, 97%, Merck Millipore, USA). The polymers used to compare the performance of the nanohybrid in EOR processes were P88 (MW = 9–12 MDa, % hydrolysis = 28), PA2 (MW = 11 MDa, % hydrolysis = 28) y P51 (MW = 10 MDa, % hydrolysis = 12). The viscosity of the crude oil was 1580 cP @ 25 °C and 135 cP @60 °C (BS&W 2% wt).

The injection brine was prepared with 33,377 ppm sodium chloride (NaCl, 99.5% pure, Merck Millipore, USA), 93 ppm potassium chloride (KCl, 99.5% pure, Merck Millipore, USA), 870 ppm magnesium chloride (MgCl_2_.6H_2_O, 99% pure, Merck Millipore, USA), and 2494 ppm calcium chloride (CaCl_2_.2H_2_O, 99% pure, Merck Millipore, USA). Ecopetrol S.A. supplied the sandstone cores. Potassium thiocyanate (30 ppm, KSCN, 99.7% pure, J.T. Baker) was used as a tracer in the retention tests.

### 2.2 Nanohybrid Synthesis

**Figure 1:**
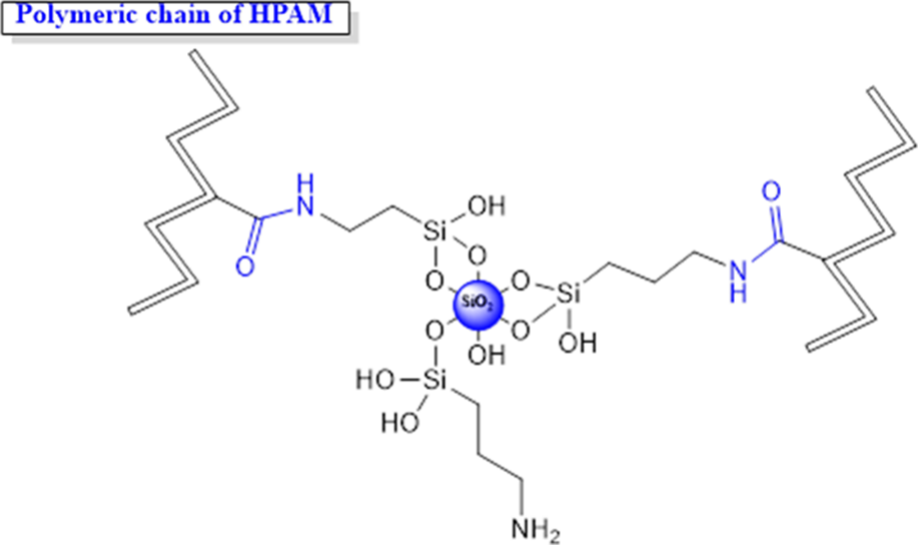
Nanohybrid structure.

The nanohybrid (NH34) was synthesized by dispersing 2 g of nSiO_2_-APTES in a THF/water solution at 400 rpm and adding 3 g of P34 powder (Figure 1). The reaction proceeded under ambient conditions for 24 h. Subsequently, NH34 was recovered via centrifugation and purified by washing with 2-propanol. The final product was then dried at 60 °C for 24 h.

### 2.3 Nanohybrid Characterization

FTIR spectra were acquired using a total attenuated reflectance platinum cell within the 4000–600 cm^–1^ range. The thermal properties of the nanohybrid, polymer, and NPs were analyzed via thermogravimetry using a TA2050 TGA analyzer (TA Instruments, Inc., USA). The samples, weighing 5 mg each, were heated from 25 to 800 °C under a nitrogen atmosphere. Finally, their thermal stability was determined following the ASTM E2550-17 (2007) standard.

**Table 1:** Petrophysical Properties of the Rock Samples Used in the Adsorption, IPV, RF, and RRF Tests (Confinement Pressure: 5516 kPa)

properties/porous media	rock 1	rock 2	rock 3	rock 4
depth, m	2364.5	2555.5–2563.5	2554.4–2430.6	2349.9
length, cm	6.08	13.49	13.58	6.65
diameter, cm	3.71	3.73	3.79	3.82
pore volume, cm^3^	11.45	23.76	23.82	15.90
porosity, %	17.90	18.90	16.20	22.00
Klinkenberg permeability, mD	292	95	95	164
injected fluid	NH34/P34	PA2	P51	P88

**Figure 2:**
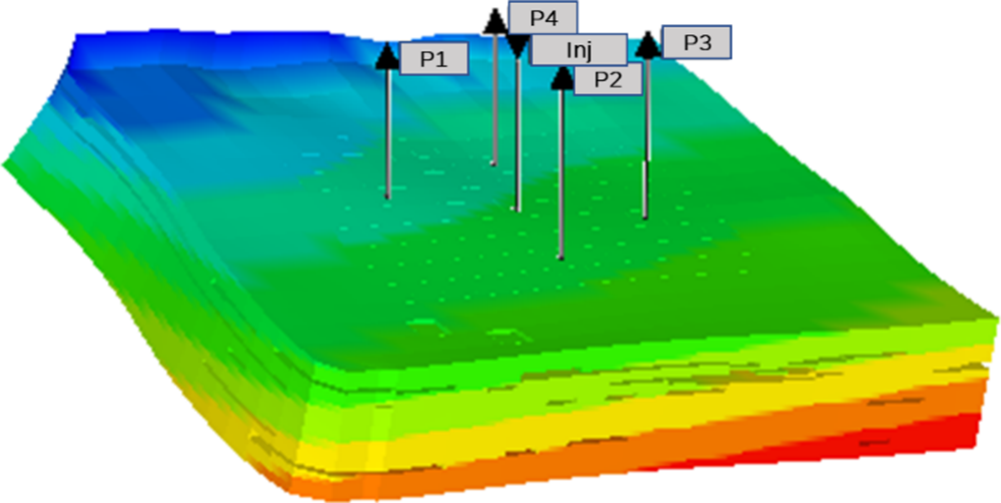
Simulation grid.

**Table 2:** Operational Conditions of the Wells

restriction	producer	injector
maximum fluid flow, m^3^/day	318	318/238
bottom hole pressure, kPa	6895	34,474

**Figure 3:**
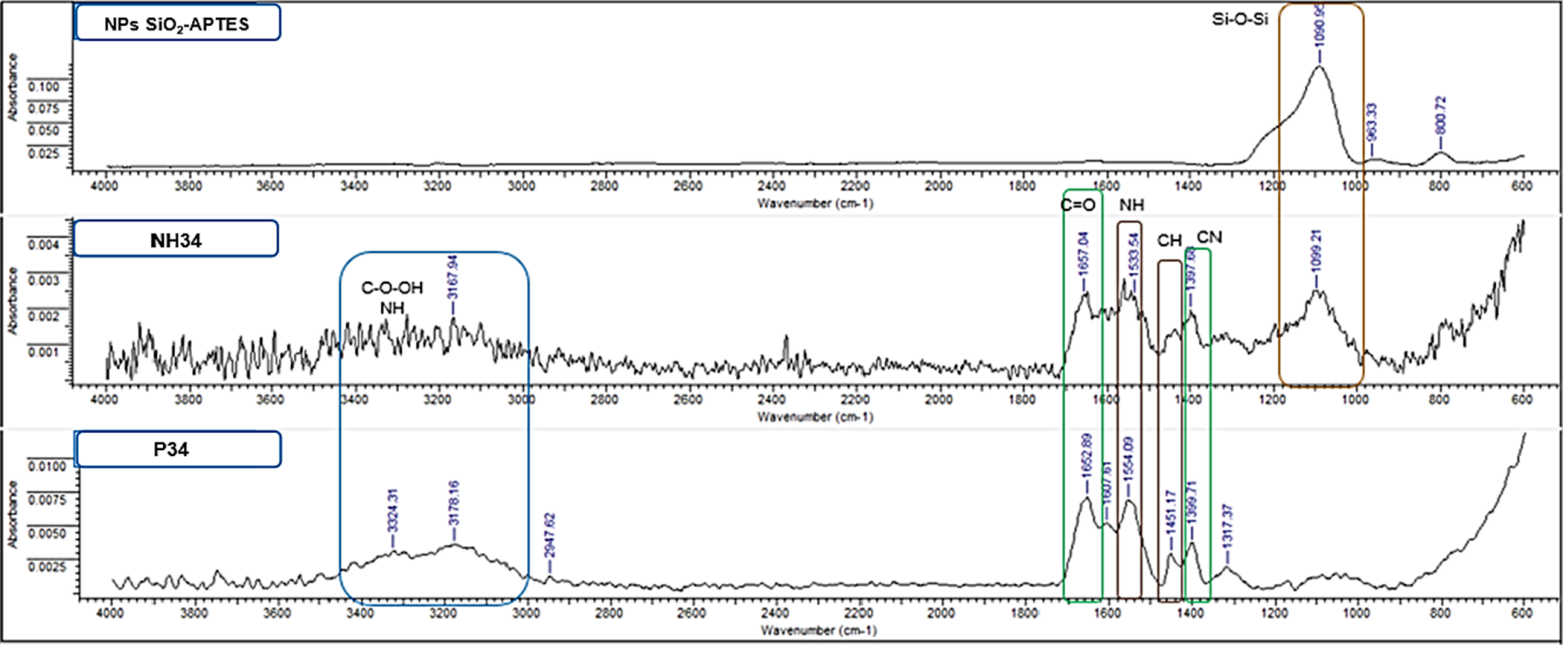
Infrared spectra of nSiO_2_-APTES, NH34, and P34.

The hydrodynamic size and ζ-potential of the NH34 and nSiO_2_-APTES were measured at 25 °C by using a Zetasizer Nano ZS 90 (Malvern Instruments Ltd., Malvern, UK). For the tests, a 100-ppm sample was dispersed in deionized water and sonicated for 15 min. pH values were then measured at 25 °C using a digital pH meter (Fisher Scientific, model AB 15 plus, Santa Barbara, CA, USA) with an uncertainty of less than ±0.05.

### 2.4 Fluid Preparation

Before use, the injection brine underwent filtration through a 5.0 μm MCE membrane (Merck Millipore, USA). Stock solutions with a concentration of 5,000 ppm were prepared by adding 5 g of either HPAM or NH34 powder to the injection water, followed by 48 h of stirring at 200 rpm. These stock solutions were subsequently diluted to the desired concentration. For rock-fluid tests, 30 ppm of KSCN was added to either the polymer solution or the nanopolymer sol.

### 2.5 Fluid–Fluid Evaluation

#### 2.5.1 Viscosity

The viscosity of the samples was measured at reservoir temperature (60 °C) in a DV3TTM rheometer (AMETEK Brookfield, USA) with a ULA adapter (μ < 100 cP, Accuracy: ±1.0%, Repeatability: ±0.2%). The concentration was varied within the range of 500–2000 ppm to establish the concentration needed to reach the target viscosity of the study field (10 cP).

#### 2.5.2 Filter Ratio (FR)

This test ensures that the polymeric solutions are free of aggregates (RF < 1.2) that could cause injectivity problems. The test was conducted by filtering 300 mL of the HPAM or nanohybrid solution through a 5 μm cellulose filter (Merck Millipore, USA). The collection times for 300, 200, and 100 mL of the filtered fluid were recorded, and FR was calculated as follows:
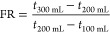
1

#### 2.5.3 Viscosity Retention Ratio

It is determined by the viscosity loss of the sample after shearing, which should not exceed 40%. The solutions were passed through narrow nozzles with inner diameters of 1/8 and 1/16 inch to induce shearing. Shearing was accomplished by subjecting the samples to pressures between 69 and 827 kPa, which were established considering the shear rate that limits the injection rate in the field (between 5000 and 30,000 s^–1^). The shear-rate values (γ̇)^49^ and the viscosity loss after shearing were calculated with the following equations:
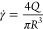
2
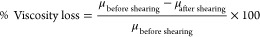
3

*Q* is the flow rate (cm^3^/s), and *R* is the capillary radius (cm).

**Figure 4:**
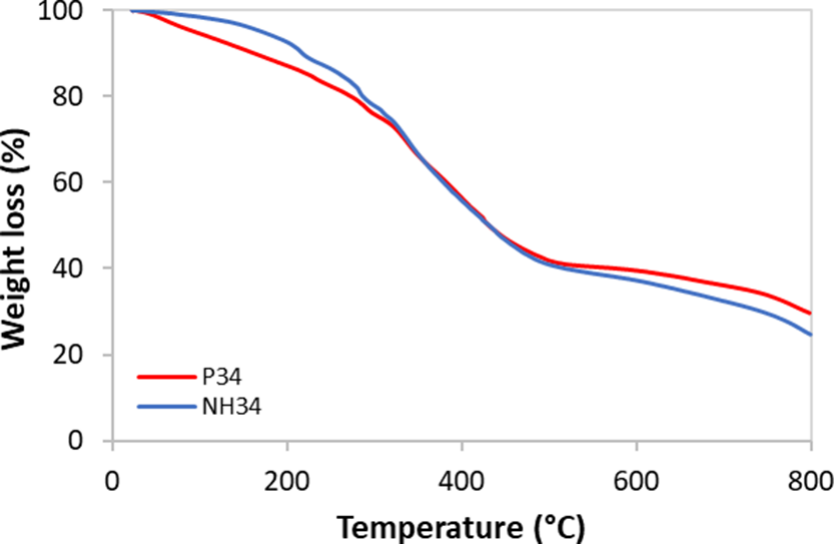
TGA profiles of P34 and NH34.

**Figure 5:**
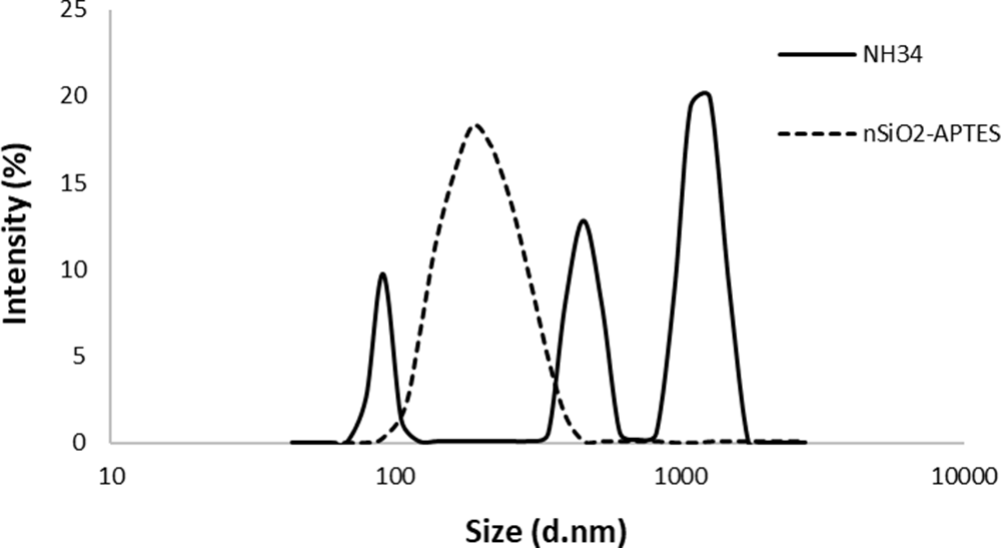
Size distribution by the intensity of nSiO_2_-APTES and NH34.

**Figure 6:**
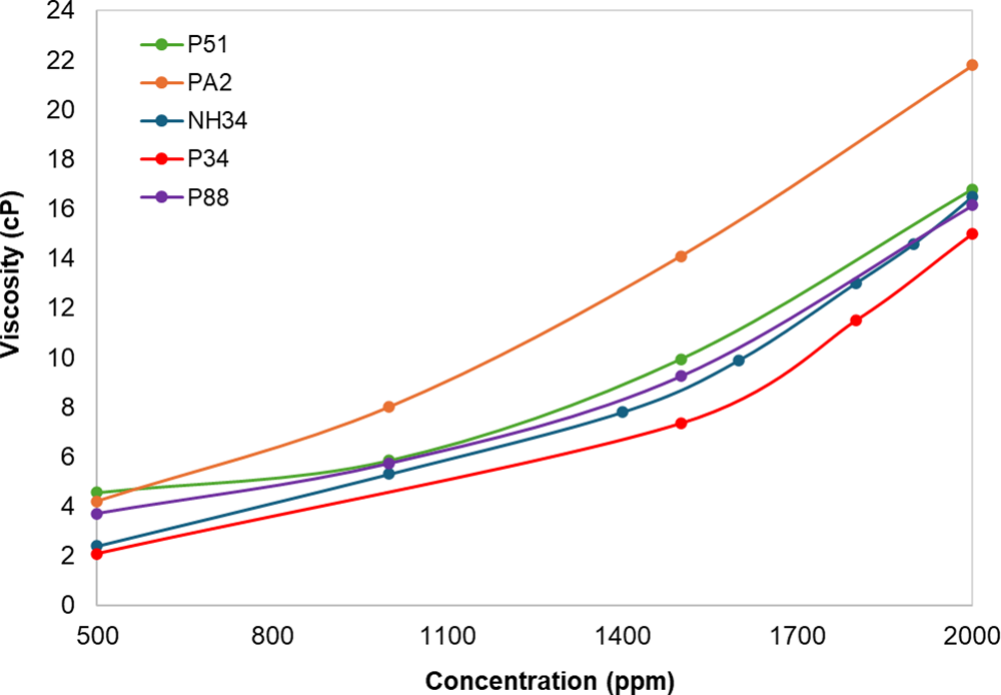
Viscosity vs concentration of PA2, P51, P88, P34, and NH34 in injection brine (TDS ∼ 35.600 ppm) at 60 °C and 7.3 s^–1^.

#### 2.5.4 Emulsion Preparation and Phase Separation Monitoring

##### 2.5.4.1 Evaluation under Producer Well Conditions

The evaluation was conducted at NH34 concentrations of 380 and 760 ppm and three^3^ water cuts (50, 70, and 90%). The initial emulsions were prepared by pouring a determined volume of crude oil into a beaker and adding brine until reaching a 30% water cut under mechanical stirring (340 rpm). The samples were stirred until total water incorporation was achieved. Before the emulsions were prepared, the crude oil and water were preheated at 85 °C for 1 h.

The desired emulsion volume was poured into a 500 mL Schott bottle, and 10 mL of brine (preheated at 85 °C) was added every 15 s under stirring (6400 rpm) to achieve the selected water cut. Then, the samples were placed in an oven at 55 °C for 5 min. Afterward, 7.5 mL of crude oil was taken for the basic sediment and water (BS&W) analysis, and the sample was placed back in the oven to measure the volume of water separated after 1, 2, 3, and 24 h. At 24 h, a crude oil sample was taken for droplet size, viscosity, and BS&W analysis. pH, turbidity, oil and grease content, and viscosity were measured in the aqueous phase.

The fluids must be preheated at 85 °C before preparing the emulsion to reduce the crude oil viscosity, obtain better dispersions and stable emulsions, reduce the interfacial tension between the dispersed and continuous phases, and provide the energy required in the emulsification process. However, the emulsion behavior was evaluated at 60 °C to simulate the surface and producer well conditions.

##### 2.5.4.2 Evaluation under Surface Fluid Treatment Conditions

This stage included evaluating the crude oil dehydration and water clarification processes using bottle tests. NH34 concentrations of 190, 380, and 570 ppm were used in the tests.

**Bottle test at surface conditions – crude oil dehydration.** Synthetic W/O emulsions were prepared using the procedure previously described. The tests were carried out at a 70% water cut (according to the tank entry condition at the treatment station). After that, 152 ppm of the emulsion breaker used in the station was added to the emulsions. The samples were shaken and placed in an oven at 55 °C. The changes in phase separation were recorded over time (1, 2, 3, and 24 h). A crude oil sample was taken for droplet size, viscosity, and BS&W analysis at 24 h. pH, turbidity, oil and grease content, and viscosity were measured in the aqueous phase.

**Bottle tests at surface conditions – water clarification.** After adding the emulsion breaker, the bottles containing different residual concentrations of NH34 (190, 380, and 570 ppm) were left standing in an oven for 1 h at 55 °C. Subsequently, 90 mL aliquots of the aqueous phase were taken from each bottle (O/W emulsion), and 6 ppm of coagulant was added. The samples were shaken vigorously for 30 s and placed in an oven at 45 °C. After 1 h, 5 mL of sample were extracted, and the oil and grease content (O&G) was measured. This procedure was repeated after 3 and 24 h. The pH and turbidity of the aqueous phases were measured after 24 h.

**Table 3:** TGA Results for P34 and NH34

sample	stage 1		stage 2		stage 3	
	weight loss, %	temperature, °C	weight loss, %	temperature, °C	weight loss, %	temperature, °C
NH34	12.2	34–235	25.7	235–365	23.6	365–563
P34	12.8	31–200	33.6	200–410	13.0	410–546

**Figure 7:**
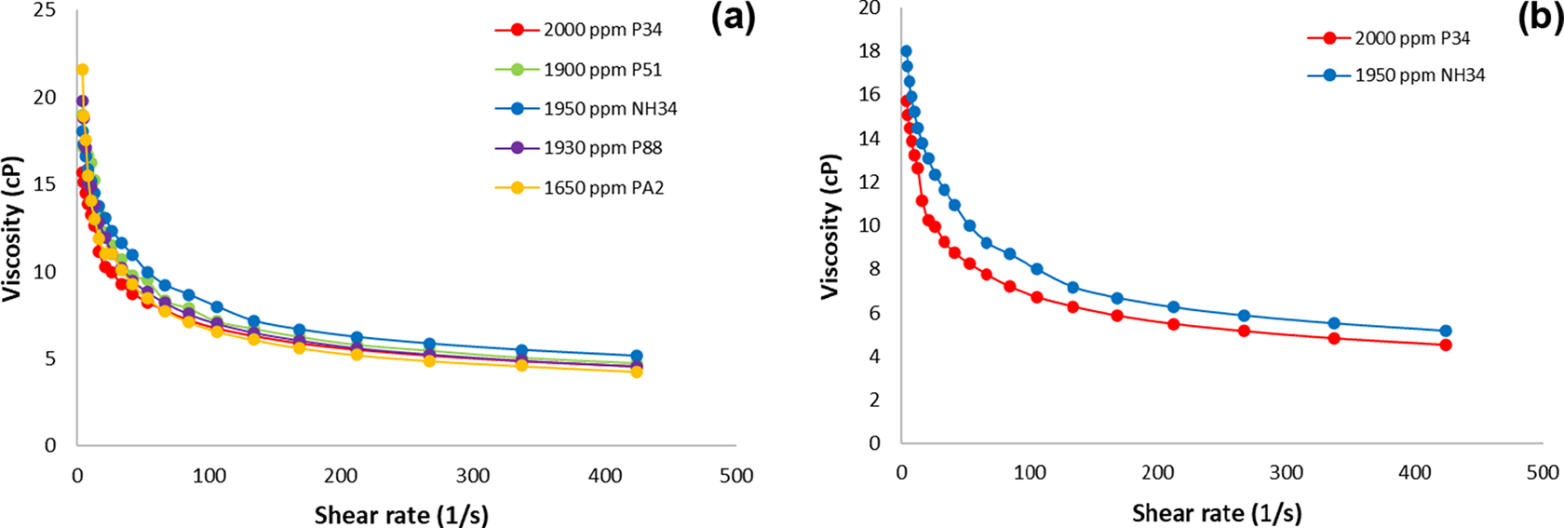
Flow curves of (a) PA2, P51, P88, P34 and NH34, and (b) P34 and NH34 in injection brine (TDS ∼ 35.600 ppm) at 60 °C.

**Figure 8:**
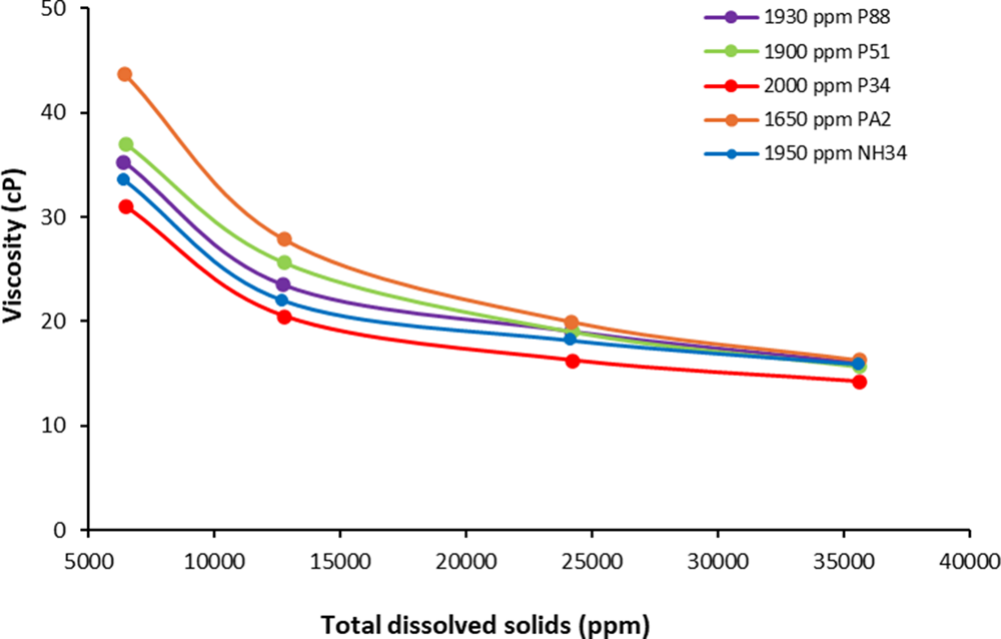
Viscosity vs TDS of PA2, P51, P88, P34, and NH34 at 60 °C and 7.3 s^–1^.

**Table 4:** Viscosity Loss of PA2, P51, P88, P34, and NH34 at 60 °C and 7.3 s^–1^

sample	1950 ppm of NH34	2000 ppm P34	1650 ppm PA2	1930 ppm P88	1900 ppm P51
TDS, ppm	viscosity loss, %				
12,742	34.5	34.0	36.3	33.4	30.8
24,158	45.9	47.6	54.4	46.0	48.6
35,573	52.7	54.2	62.8	54.6	57.6

**Figure 9:**
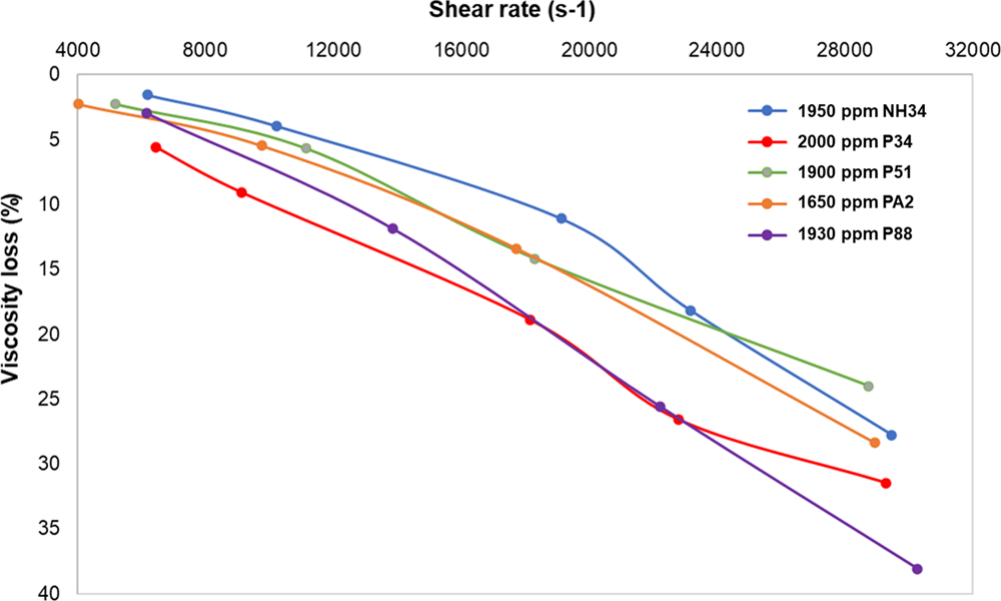
Viscosity loss due to mechanical degradation of P88, PA2, P51, P34, and NH34 in injection brine at 60 °C.

**Table 5:** Filter Ratio of PA2, P51, P34, and NH34

sample	concentration, ppm	filter ratio
NH34	1950	1.19
P34	2000	1.28
P51	1900	1.09
PA2	1650	1.14
P88	1930	1.00

### 2.6 Rock-fluid Evaluation

First, the rock samples were vacuumed and saturated with an injection brine. Brine was injected at varying flow rates (between 0.067 and 0.5 mL/min) to calculate the absolute permeability, with the pressure differentials recorded at each stage. To determine the RF and RRF, the polymer solutions or the nanohybrid sol were injected at the identical flow rates employed to determine absolute permeability, followed by brine injection. Stable pressure differentials were recorded, and the RF and RRF values were calculated from eqs 4 and 5.^50^ The shear rate (γ̇) in porous media was determined using eqs 6 and 7(51,52):
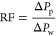
4
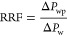
5
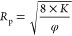
6
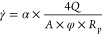
7where Δ*P*_w_ is the pressure drop during brine injection, Δ*P*_wp_ is the pressure drop during brine injection after polymer flooding, Δ*P*_p_ is the pressure drop during polymer or nanopolymer sol injection, *Q* is the flow rate (cm^3^/min), *A* is the surface flow area of the porous media (cm^2^), φ is porosity (fraction), *K* is the absolute permeability (cm^2^), *R*_p_ is the porous radius (cm), and α is the formation shape factor which is assumed 1 (dimensionless) for the sandstone plugs.

The material balance method was employed to quantify adsorption and the inaccessible pore volume (IPV). Each sample containing 30 ppm of KSCN tracer was injected until the *C*/*C*_o_ ratio in the effluents equaled 1. Subsequently, brine was injected until the effluent polymer concentration approached zero. All fluids were injected at a rate of 0.067 mL/min. Effluents were collected for tracer, HPAM, and NH34 concentration determination via UV–vis analysis (DR5000, Hach, USA). For these measurements, two 1 mL aliquots of the effluents underwent distinct treatments: one for the KSCN measurement using iron chloride hexahydrate and the other for P34 and NH34 concentration determination using sodium hypochlorite and glacial acetic acid. This procedure was replicated for the second batch of either the P34 or the NH34 solution. IPV and adsorption were calculated using the following equations:
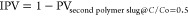
8
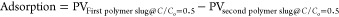
9

*C* is the polymer concentration in the effluent, and *C*_o_ is the initial polymer concentration.

**Figure 10:**
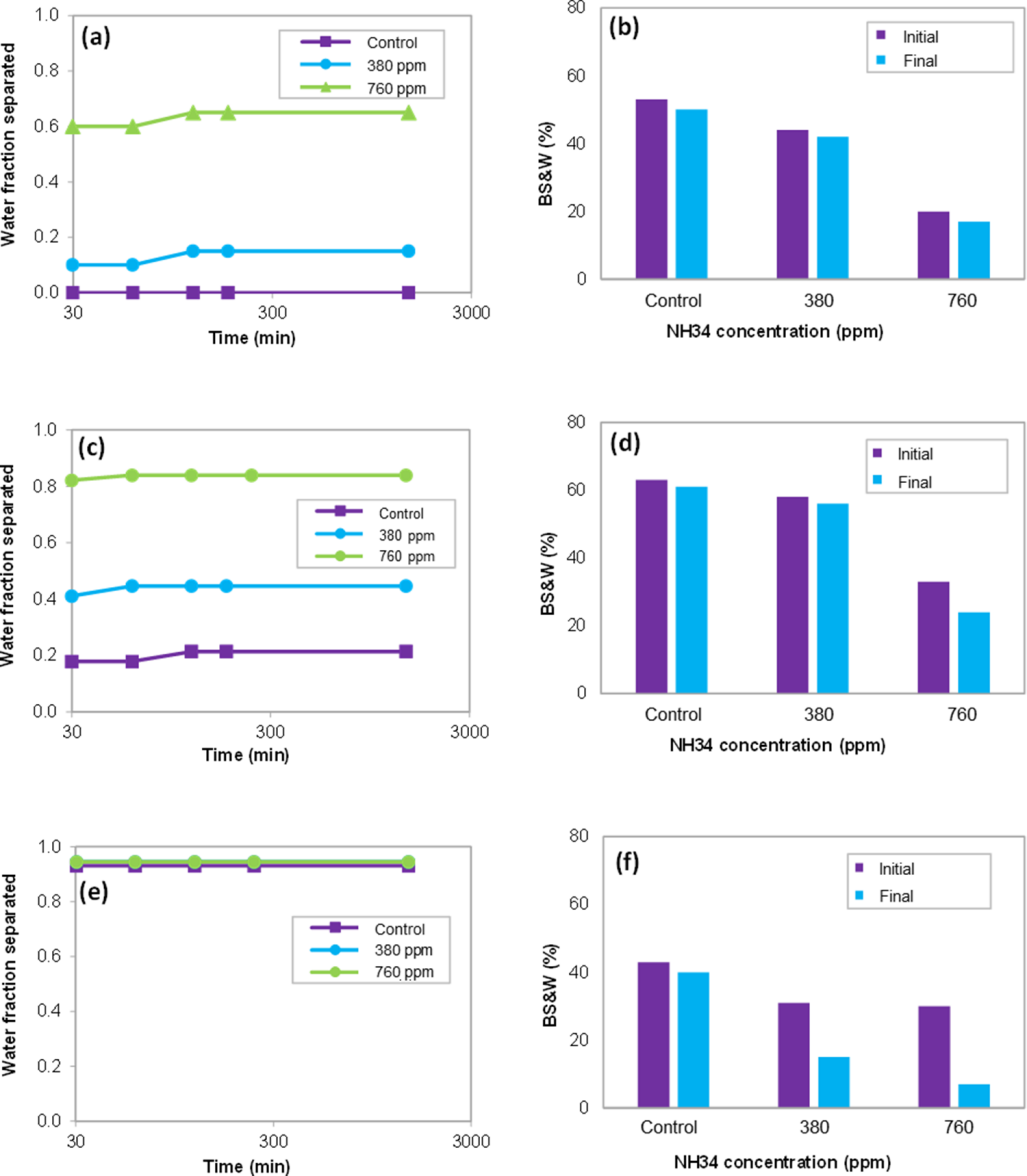
Water fraction separated and % BS&W of the oleic phase at NH34 concentrations of 0 (control), 380, and 760 ppm and water cuts of (a,b) 50, (c,d) 70, and (e,f) 90%.

**Figure 11:**
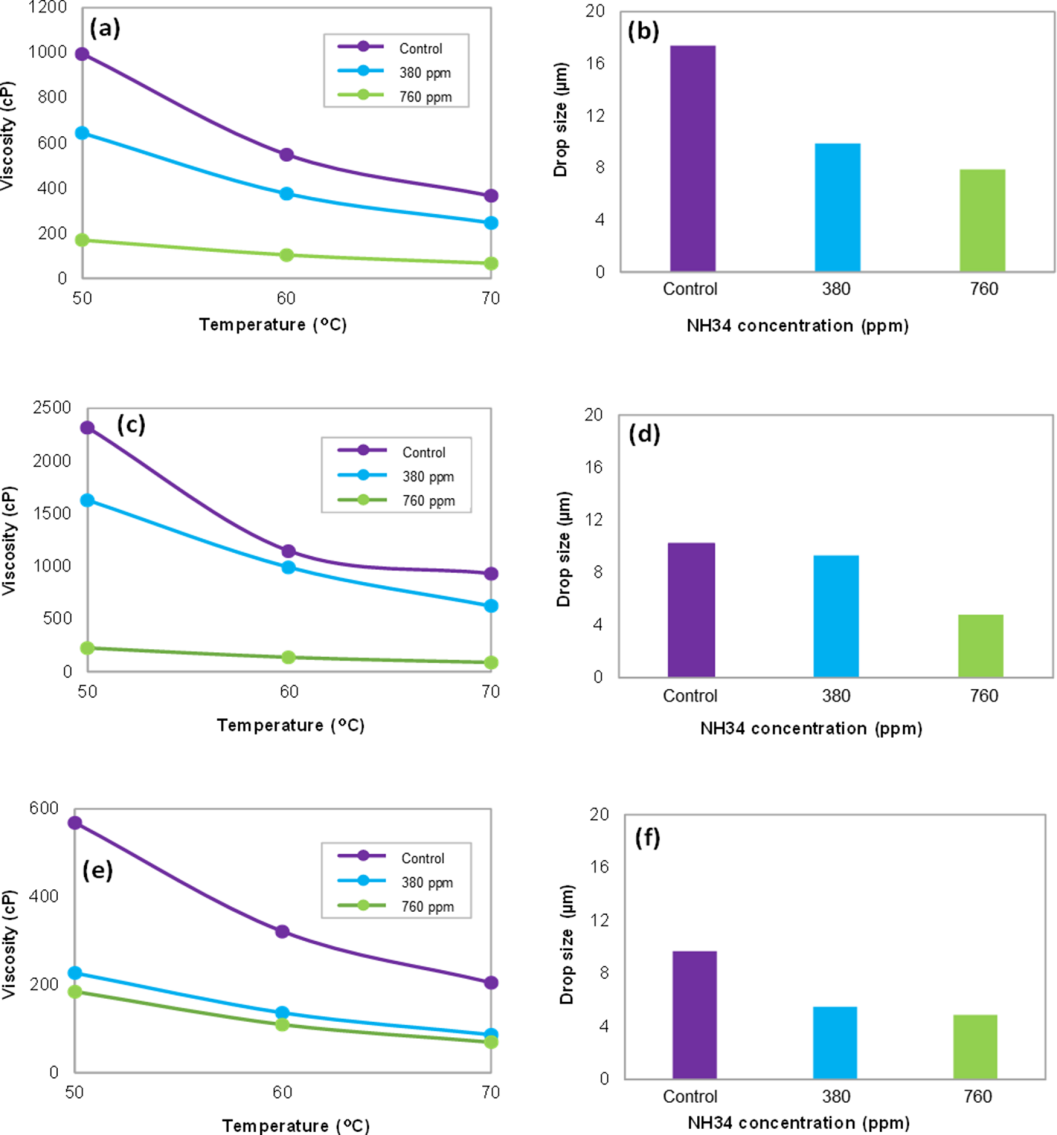
Viscosity (0–100 s^–1^) and droplet size of the oleic phase at NH34 concentrations of 0, 380, and 760 ppm and water cut of (a,b) 50, (c,d) 70, and (e,f) 90%.

**Table 6:** Adsorption and IPV of PA2, P88, P51, P34, and NH34 at 60 °C

sample	adsorption, μg_product_/g_rock_	IPV, %	rock sample
NH34	83,7	25	1
P34	20,2	19	1
P51	40,5	19	3
PA2	191,3	22	2
P88	45,0	25	4

Table 1 presents the properties of the reservoir rock samples. The characterization of these properties followed the protocols outlined by McPhee and Arthur.^53^ All experiments were conducted at 60 °C, corresponding to the reservoir temperature of the Colombian field selected for assessing the performance of the synthesized nanohybrid. The average mineralogical composition of the rock samples is 67.5% quartz and 26% clay minerals, which in the order of abundance corresponds to kaolinite (82.5%), iIllite (16.5%), and interstratified (1.0%).

Prior to injection, both the polymer solutions and nanopolymer sols underwent filtration and preshearing. For the preshearing step, 300 mL of sample were pressurized with nitrogen and passed through a capillary with an inner diameter of 1/8 in.

### 2.7 Numerical Simulation

The numerical simulation was carried out with commercial software (CMG STARS). An inverted 5-spot injection pattern centered on the simulation grid was defined to evaluate the effect of the injection of the nanohybrid and the four^4^ commercial polymers (P34, P88, P51, and PA2) (Figure 2). The pattern covers an area of 20 acres, with a pore volume of 1.3 Mm^3^ (8.05 Mbbl) and an original oil-in-place (OOIP) volume of 0.8 Mm^3^ (5.27 Mbbl). The operational conditions for the producer and injector wells are presented in Table 2.

To predict the behavior of nanohybrid injection, the simulation model underwent an initial stage of water injection as a secondary recovery method. This established a baseline to assess the performance of the injected chemicals. Irreversible polymer adsorption was considered in the simulations.

## 3 Results and Discussion

### 3.1 Nanohybrid Characterization

#### 3.1.1 FTIR

FTIR spectra of nSiO_2_-APTES NPs, P34, and NH34 are compared to confirm the polymer’s covalent bonding with the NPs (Figure 3). The characteristic peaks confirming the hybrid formation include stretching vibration of −NH and −OH at 3324 cm^–1^.^54,55^ Additional stretching vibrations observed correspond to the −CH_2_ (2947 cm^–1^), −C=O (1662 cm^–1^), C–N (1307 cm^–1^), and Si–O–Si (1060 cm^–1^)^56^ groups.

#### 3.1.2 TGA Results

Figure 4 depicts the TGA profiles of the NH34 nanohybrid and P34 polymer. Weight loss in both materials occurs in three stages (Table 3). The first stage occurs between 30 and 235 °C and is attributed to the samples’ loss of water or volatile solvents. In this temperature range, P34 exhibited a weight loss of 12.8%, while NH34 showed a slightly lower weight loss of 12.2%. The second stage, occurring between 235 and 410 °C, is assigned to the thermal decomposition of the carboxylate and amide groups within the P34 polymer. The weight loss in this stage was 33.6 and 25.7% for P34 and NH34, respectively. The final stage (*T* > 410 °C) was assigned to the decomposition of C–C bonds in the P34 polymer structure.

In our previous work,^57^ a weight loss of 2.8% for nSiO2-APTES between 350 and 600 °C was reported, which was attributed to the thermal decomposition of the aminopropyl groups. However, when compared to the weight losses reported for P34 and NH34 under the same conditions, it is not considered significant.

#### 3.1.3 Particle Size and ζ-Potential

Figure 5 reports the DLS results of nSiO_2_-APTES and NH4 in deionized water. While the NP size distribution appears monomodal, with an average size of 200 nm, the nanohybrid exhibits a trimodal size distribution. The peak at approximately 100 nm is associated with the unmodified nSiO_2_-APTES present in the sample. Meanwhile, the peaks observed between 430 and 490 nm and between 1250 and 1450 nm correspond to NH34 particles and particle aggregates, respectively. These aggregates form due to intermolecular associations of the P34 chains facilitated by hydrogen bonding

The ζ-potential of nSiO_2_-APTES is +26.2 mV at pH 7, suggesting that the NP dispersion could potentially be unstable. Upon the NPs-polymer bonding, the potential changes sign (−36.9 mV), due to the negative charges in the HPAM backbone. This value shows that the NH34 solutions are stable.

### 3.2 Fluid–Fluid Evaluation

#### 3.2.1 Viscosity Measurements

The viscosities of all samples as a function of concentration are displayed in Figure 6. The concentration needed to obtain the target viscosity of 10 cP for NH34 is 1950 ppm, and for P34 is 2000 ppm. The nanohybrid would decrease the required product injection amount because the NPs are covalently bonded with the polymer chains forming a network-like structure which creates physical barriers that resist polymer chain alignment or disentanglement under shear forces.^32,36,41,58,59^

Experimental evaluations previously performed for the selected field showed that the required concentrations of the polymers P88, P51, and PA2 to achieve the same viscosity were >2000, 1950, and 1650 ppm, respectively. Although PA2 demands a lower concentration, it is not included in the comparison due to its high adsorption in porous media (Table 6). The viscosity losses reported in Figure 9 were considered to determine the target concentration for each sample.

The flow curves of PA2, P51, P88, P34, and NH34 are listed in Figure 7. All samples show pseudoplastic behavior. However, the nanohybrid exhibits slightly higher viscosities at shear rates exceeding 10 s^–1^ compared to those of all the polymers. This behavior arises from the interaction between NPs and the polymer, which enhances its resistance to shear forces.

Figure 8 shows that the viscosity of all samples decreases as the TDS content increases. This effect occurs because the presence of counterions (such as Na^+^, K^+^, Ca^2+^, and Mg^2+^) neutralizes electrostatic interactions along the polymer backbone. As a result, the polymer chains can fold, leading to a decrease in the hydrodynamic size of these macromolecules. This decrease in chain size also reduces interactions with neighboring chains, impacting the viscosity of the solutions. The viscosity loss of all samples is reported in Table 4. The nanohybrid exhibited better resistance due to the NP-polymer bonding, which reduced the available interaction sites of the counterions with the polymer backbone.

#### 3.2.2 Mechanical Degradation Tests

All products exhibit viscosity losses below 40% at all shear rates (Figure 9). However, the nanohybrid shows lower viscosity losses than all the polymers evaluated at shear rates below 22,000 s^–1^ (common in injection wells). Beyond this shear rate, only P51 demonstrates better performance than NH34. The viscosity reduction of all polymer solutions by mechanical forces is attributed to chain alignment, entanglement, or chain scission.^60^ The better performance of NH34 is attributed to the NP-polymer interaction, which modifies their hydrodynamic configuration, improving its shear resistance.

#### 3.2.3 Filterability Ratio

The FR should be less than 1.2 if the solutions were appropriately prepared. Table 5 reports the FR values for the nanohybrid and polymers P88, PA2, P51, and P34. These results show that all assessed samples, except P34, are unlikely to cause plugging issues when injected into the porous media under these conditions.

#### 3.2.4 Emulsion Behavior

##### 3.2.4.1 Evaluation under Producer Well Conditions

The results show lower incorporation of the aqueous phase in the oleic phase (Figure 10) and a reduction in droplet sizes (Figure 11b,d,f) as the residual concentration of the nanohybrid increases. This behavior is ascribed to the increased viscosity of the aqueous phase, reducing its incorporation into the oleic phase (Figure 11a,c,e). These results are consistent with those previously obtained for polymers P34, P51, and P88 (PA2 was not evaluated due to its high adsorption in the porous media, Table 6).

**Figure 12:**
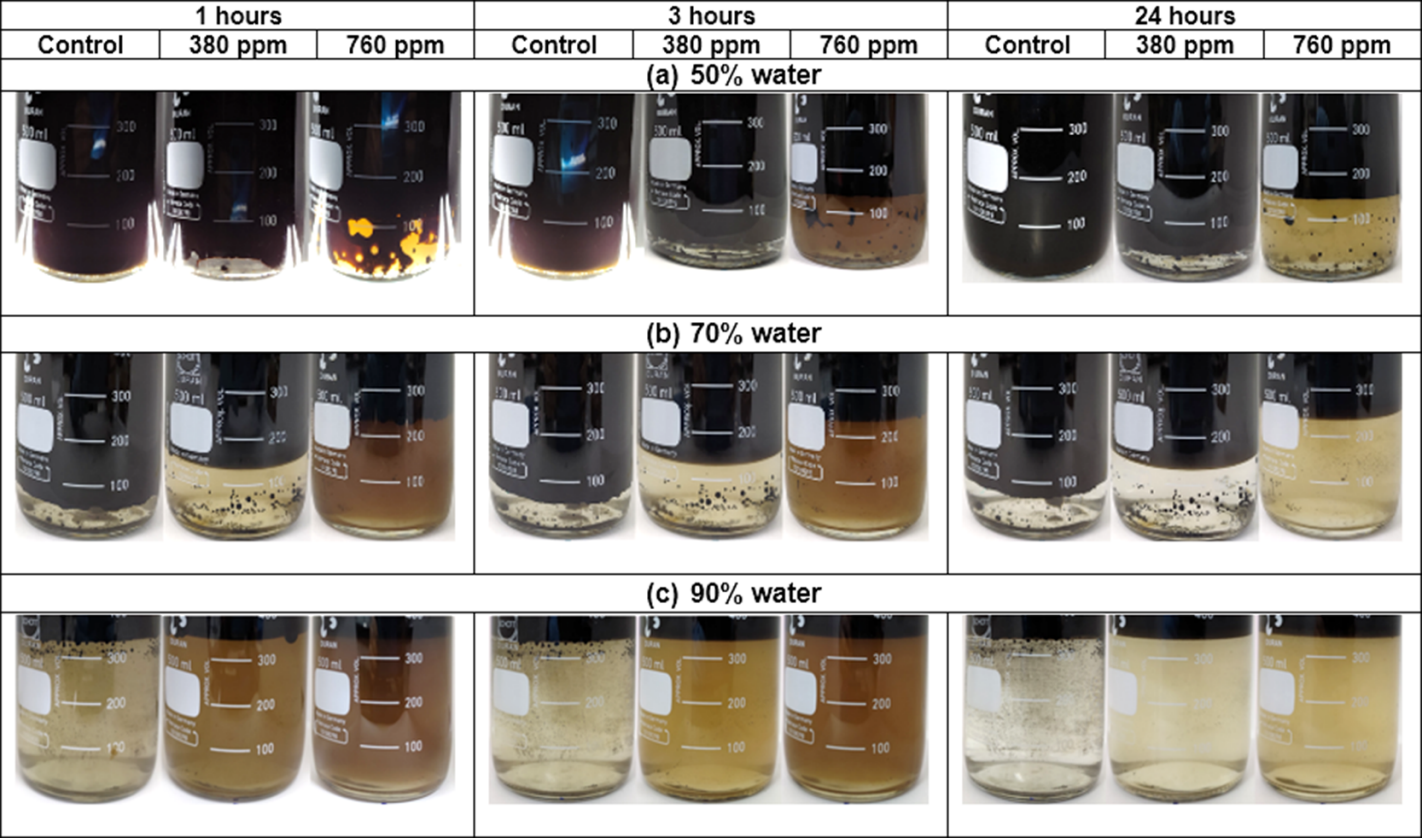
Water fractions separated after 1, 3, and 24 h at NH34 concentrations of 0, 380, and 760 ppm, and water cut of 50, 70, and 90%.

**Figure 13:**
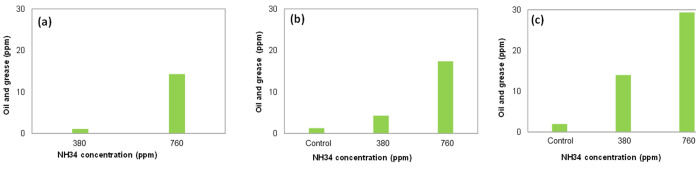
Oil and grease content in aqueous phases at NH34 concentrations of 0, 380, and 760 ppm, and water cut of (a) 50%, (b) 70%, and (c) 90% after 24 h.

**Figure 14:**
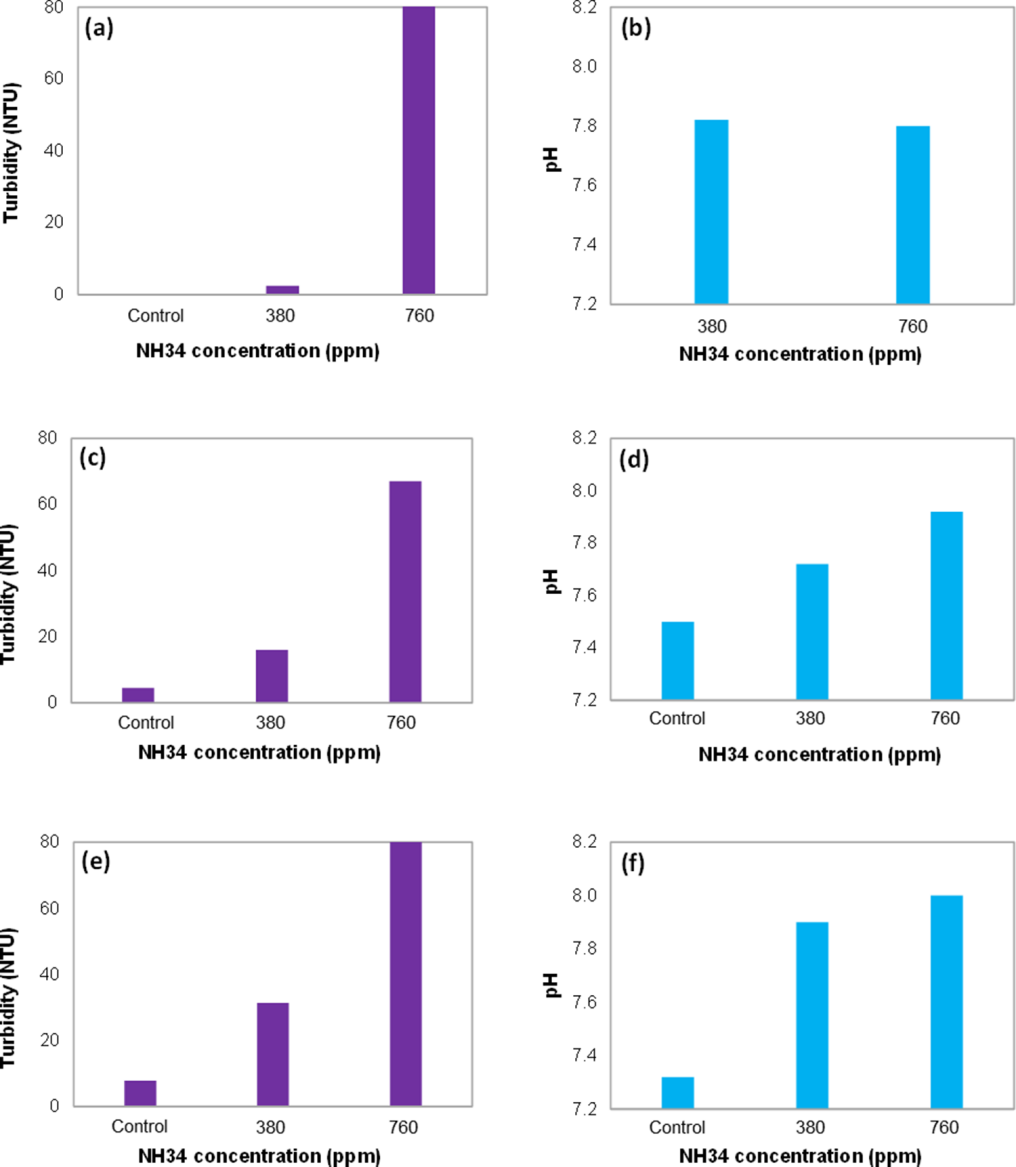
pH and turbidity values of the aqueous phases (inverse emulsion) at residual NH34 concentrations of 0, 380, and 760 ppm and a water cut of (a,b) 50%, (c,d) 70%, and (e,f) 90% after 24 h.

**Figure 15:**
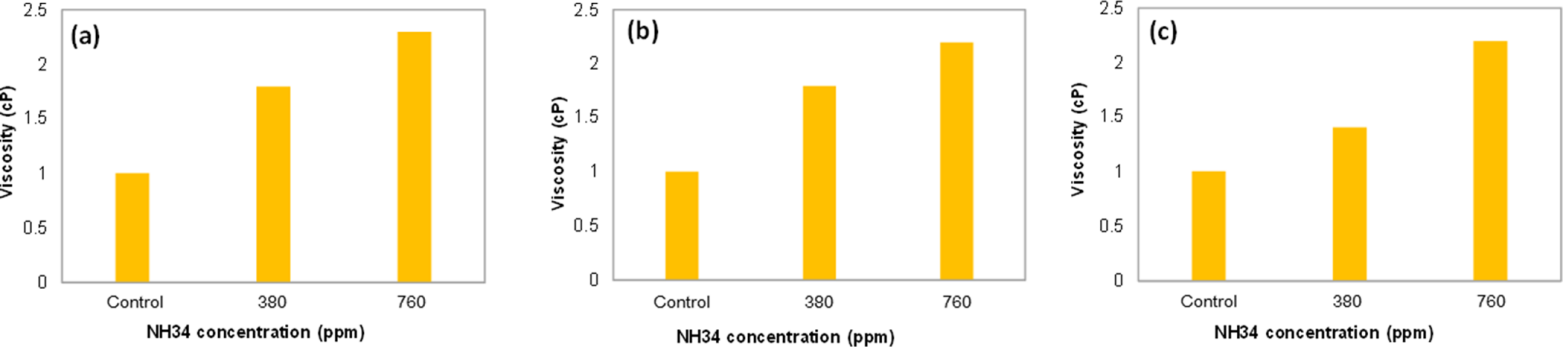
Viscosity of the aqueous phases (0–100 s^–1^, inverse emulsion) at NH34 concentrations of 0 (control), 380, and 760 ppm, water cut of (a) 50%, (b) 70%, and (c) 90%, 60 °C and 7.3 s^–1^ after 24 h.

Phase separation images are presented in Figure 12. The quality of the water (turbidity and oil and grease content) drained by gravity is affected by the increase in the residual concentration of the nanohybrid (Figures 13 and 14). This effect is assigned to the increased viscosity of the aqueous phase, preventing the coalescence of crude oil droplets (Figure 15). Understanding this effect helps to define the conditions required for the chemical and gravitational separation units in the water treatment facilities (residence times, demulsifier concentration, geometries, etc.).

##### 3.2.4.2 Evaluation of Surface Fluid Treatment Conditions

**Figure 16:**
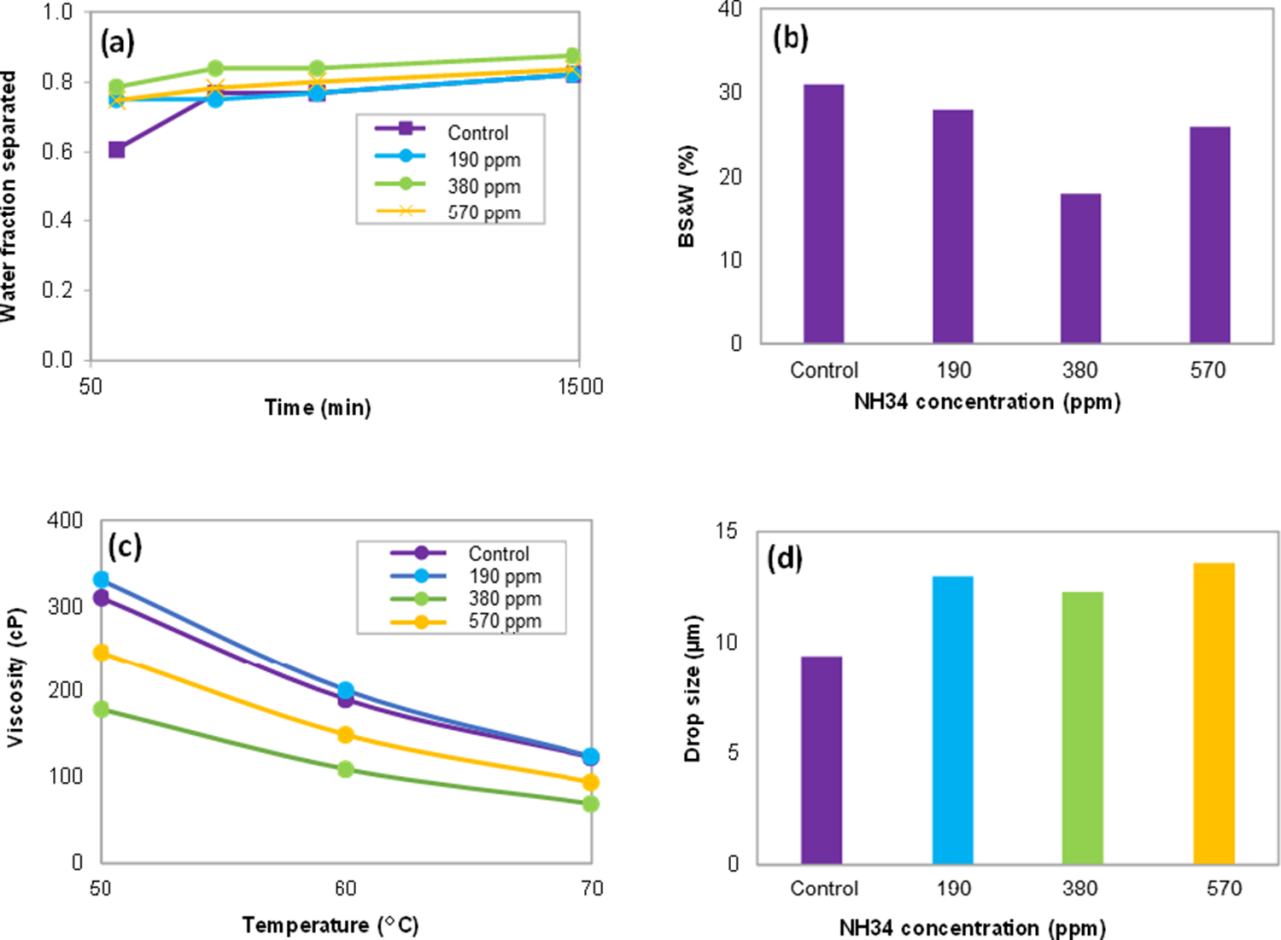
Results of (a) water fraction separated, (b) % BS&W, (c) viscosity (0–100 s^–1^), and (d) droplet size of the oleic phase at residual NH34 concentrations of 0 (control), 190, 380, and 570 ppm, and 70% water cut.

**Bottle tests under crude oil dehydration conditions.** The phase separation rate increases as the residual NH34 concentrations increase (Figure 16a). Furthermore, compared to the control sample, the %BS&W values are reduced for the samples with 190 and 380 ppm of NH34. The reduction in the water content matches the decrease in the viscosity values (Figure 16b,c).

**Figure 17:**
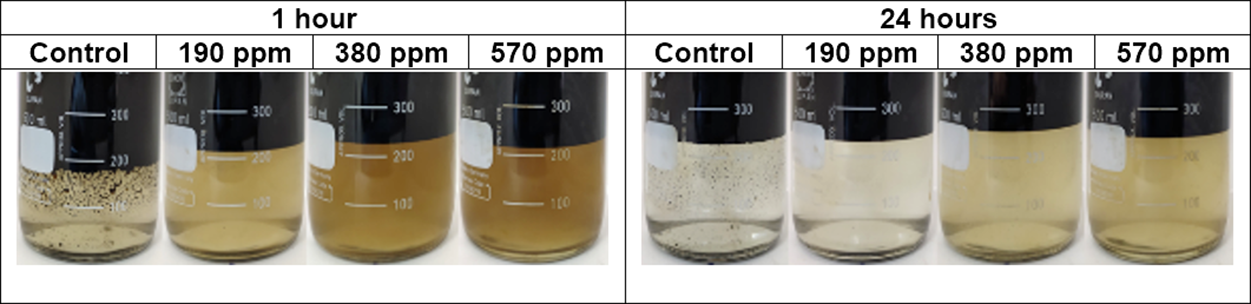
Water fractions were separated at residual NH34 concentrations of 0, 190, 380, and 570 ppm, and 70% of the water was cut after 1 and 24 h.

**Figure 18:**
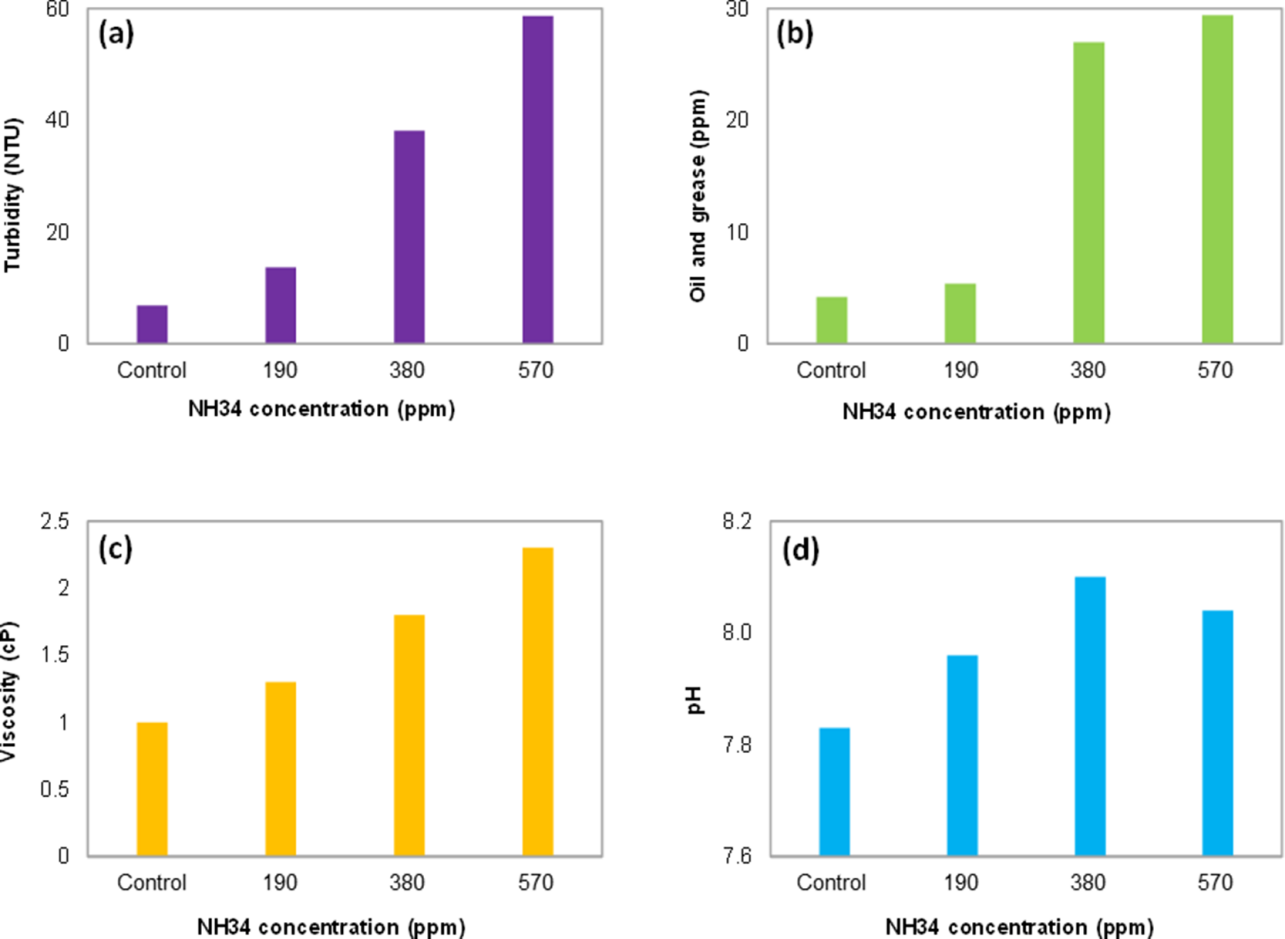
Results of (a) turbidity, (b) oil and grease, (c) viscosity (0–100 s^–1^), and (d) pH of the aqueous phase (reverse emulsion) at residual NH34 concentrations of 0, 190, 380, and 570 ppm and 70% water cut.

The images of the water fractions separated in these tests are shown in Figure 17. The water quality (turbidity and oil and grease content, Figure 18a,b) is affected by the increase in the residual concentration of the nanohybrid. This effect is caused by the increase in the viscosity of the aqueous phase that prevents the crude oil droplets from coalescing (Figure 18c). The pH values do not change significantly, avoiding the change or possible affectation of the chemistry used in the dehydration process (Figure 18d).

##### 3.2.4.3 Evaluation of Surface Fluid Treatment Conditions

**Figure 19:**
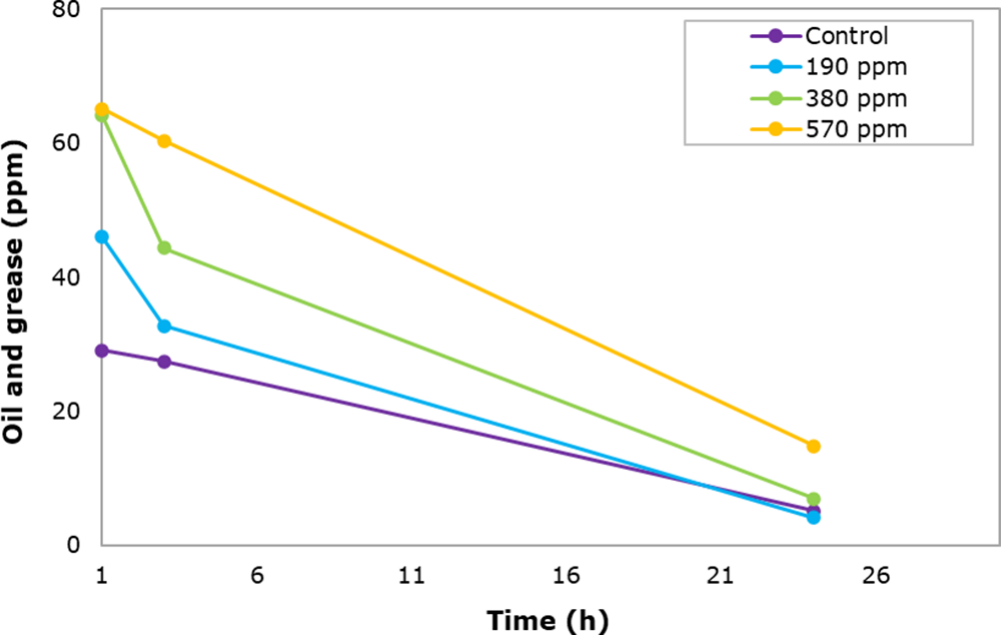
Oil and grease contents of the aqueous phase (inverse emulsion) over time at residual NH34 concentrations of 0, 190, 380, and 570 ppm and 45 °C.

**Bottle tests under water clarification conditions.** The oil and grease content of the aqueous phase (O/W emulsion) remains below 6 ppm in all samples, except for the 570 ppm sample, which had a final value of 12.4 ppm after 24h (Figure 19). The oil and grease content for polymers P51 and P88 remained below 5 ppm, with removal percentages exceeding 93%.^61^

**Figure 20:**
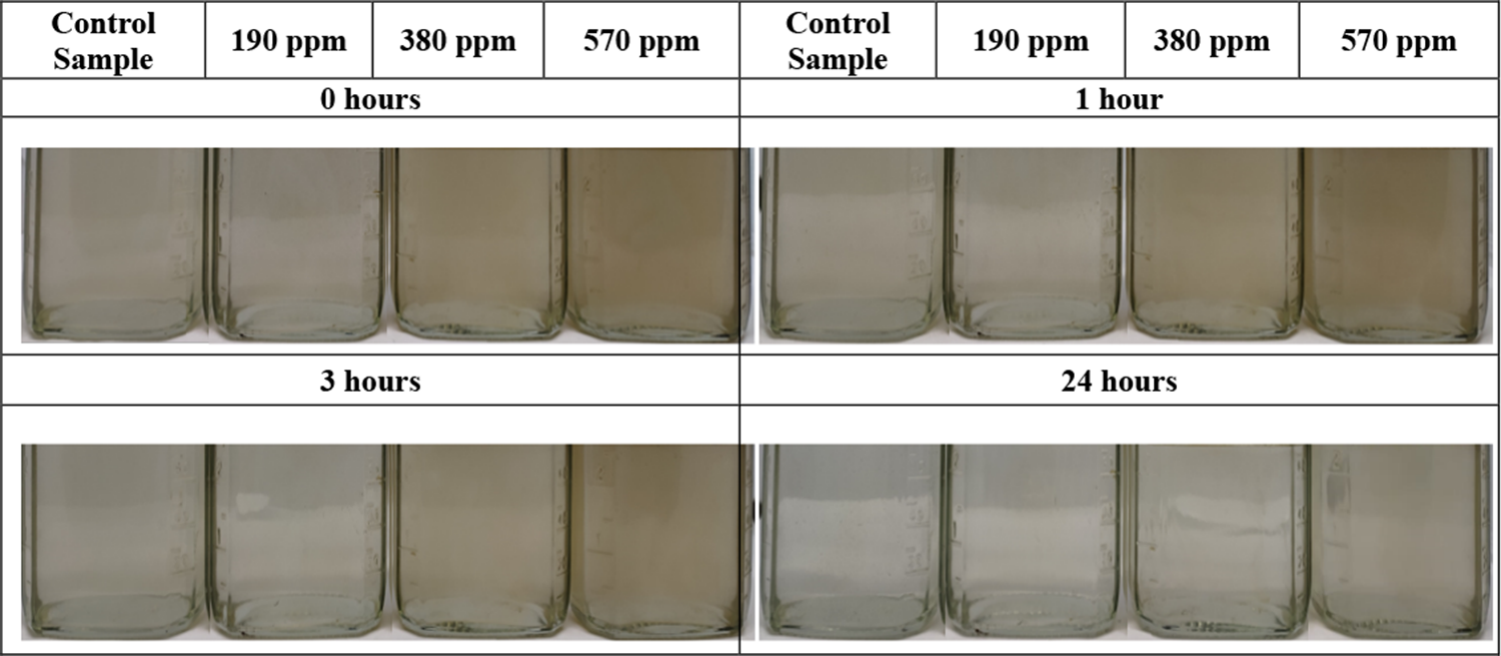
Water clarification at 45 °C and residual NH34 concentrations of 0, 190, 380, and 570 ppm were obtained after 24 h.

**Figure 21:**
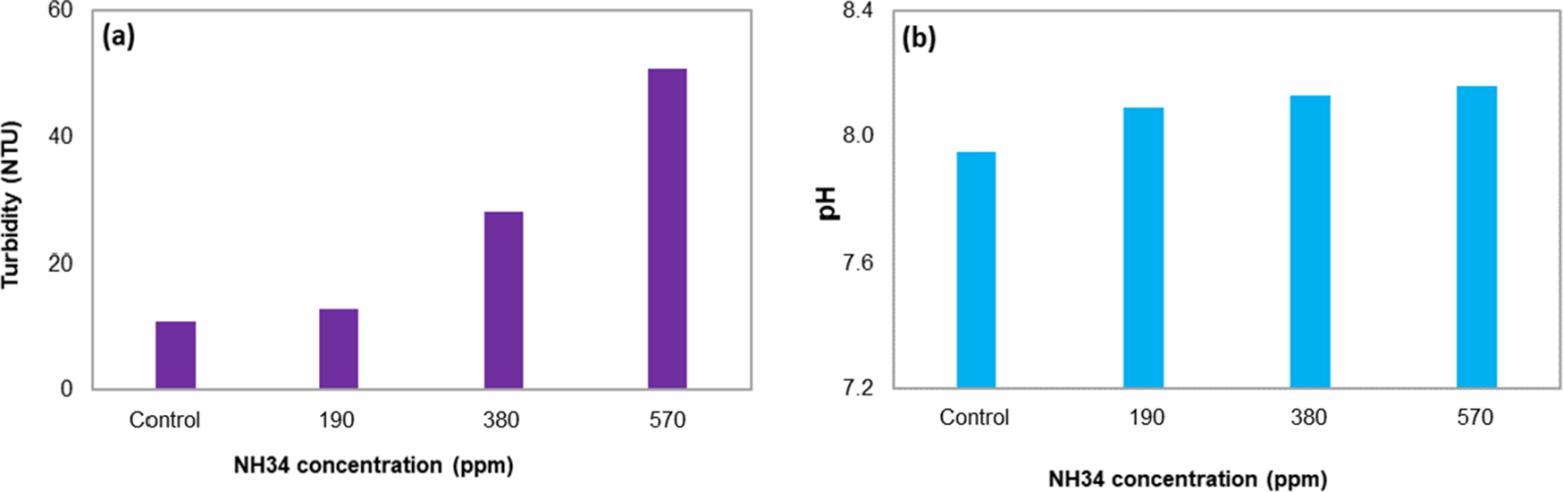
Results of (a) turbidity and (b) pH of the aqueous phase (inverse emulsion) at 45 °C and residual NH34 concentrations of 0, 190, 380, and 570 ppm after 24 h.

Photographs of the bottles used in the clarification stage are shown in Figure 20. Similar to the previous stages, residual NH34 increases the water turbidity (Figure 21a) but does not change its pH (Figure 21b).

### 3.3 Rock-Fluid Evaluation

#### 3.3.1 Adsorption, IPV, RF, and RRF

The adsorption and IPV values for PA2, P88, P51, P34, and NH34 at 500 ppm are reported in Table 6. The adsorption of the PA2 polymer is approximately 4 times higher than those of P51 and P88 and 2 times higher than that of NH34. The adsorption of the nanohybrid is 2 times higher than that of P51 and P88 and 4 times higher than that of P34, ascribed to the electrostatic attraction between the positively charged nSiO_2_-APTES and the negatively charged porous media. According to the literature, IPV values were between 19 and 25%, which is normal.^62,63^

**Figure 22:**
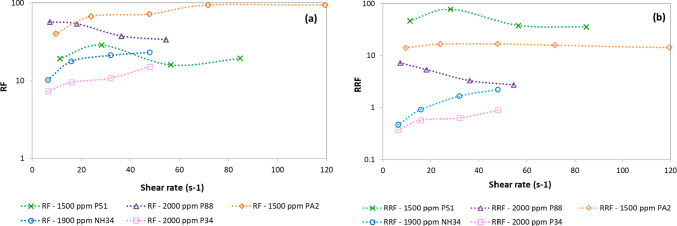
(a) RF and (b) RRF of 1500 ppm P51, 2000 ppm P88, 1500 ppm PA2, 2000 ppm P34, and 1900 ppm of NH34.

RF and RRF values for the NH34 nanohybrid and the evaluated polymers are presented in Figure 22a,b. For PA2, P34, and NH34, RF and RRF values gradually increase as the shear rate increases (eq 2). The increase in the injection rate of polymers and the nanohybrid produces elastic deformation of their molecules driven by hydrodynamic forces. This leads to an increase in the effective viscosity and RF. The increase in RRF values is attributed to the mechanical entrapment of polymer or nanohybrid molecules in the pore throats and their adsorption in the porous media.

In contrast, for the P88 polymer, the increase in the shear rate decreases the RF and RRF values. The increase in the water injection velocity (or shear rate) after chemical injection reduces RRF due to the removal of the P88 polymer molecules retained in the porous media.

### 3.4 Numerical Simulation

**Figure 23:**
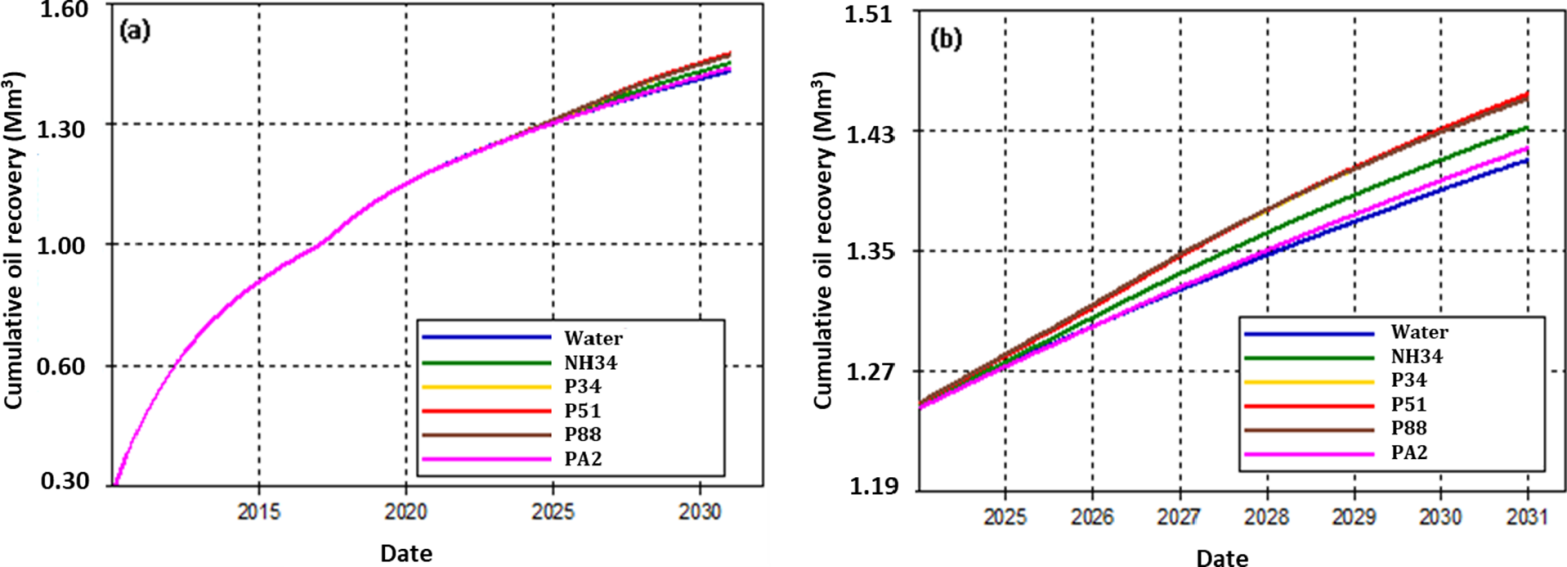
(a) Cumulative oil production by the injection of a 0.3 PV chemical slug and (b) zoom-in of the oil production in the past few years.

Figure 23 presents the cumulative oil production after injecting 0.3PV of chemicals. The polymers P51 and PA2 showed injectivity problems, achieving only 0.215 and 0.228 PV injection, respectively. The highest cumulative oil production is obtained with P51, while polymer PA2 achieved the lowest incremental oil production compared to water injection. Considering that the injection viscosity was the same for all polymers, the difference in the production increments is due to rock-fluid interactions such as adsorption, IPV, and RRF. Higher RRF of the injected chemical leads to a greater incremental oil recovery. However, high RRF values result in injectivity losses, as observed with P51 and PA2.

**Figure 24:**
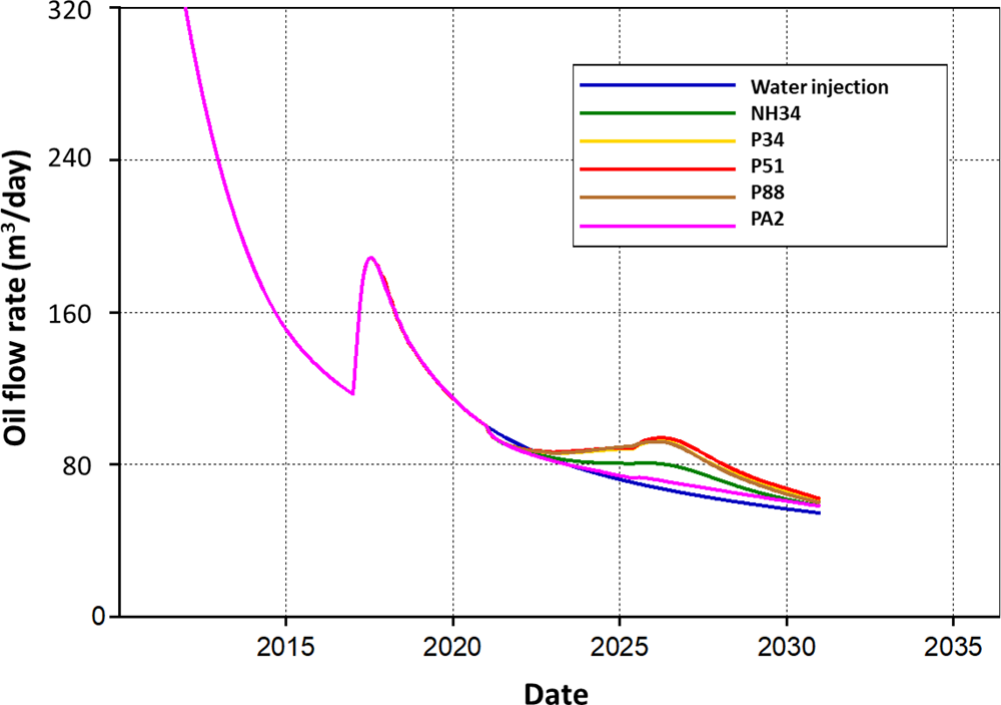
Oil production rate vs time.

Regarding the behavior of oil production rates, it is observed that the injection of chemicals smooths the decline in the production curve (nanohybrid) and even reverses this decline (P51, P34, and P88; Figure 24). The better performance of polymers can be associated with the higher RRF values obtained in the laboratory. However, these values are uncertain as they are higher than those reported for these polymers. Additionally, it should be noted that current commercial simulators fail to represent all phenomena occurring in porous media for the injection of nanohybrids and, in general, nanofluids (i.e., filtration effects). This represents a limitation in the numerical simulation of these technologies.

During chemical injection, a decrease in the oil production flow is observed at the beginning of injection due to the reduction in injection flow rates for water and chemicals (from 318 to 238 m^3^). Polymers P34, P51, and P88 changed the behavior of the oil flow positively, while NH34 flattened the decline curve. On the other hand, PA2 slightly increases the oil production flow due to its high adsorption in the porous media. Table 7 presents the incremental recovery factors by the injection of polymers and the nanohybrid compared to water injection.

**Table 7:** Incremental Recovery Factors by the Injection of Polymers and Nanohybrids Factors

polymer	incremental oil production, Mm^3^	recovery factor, %
NH34	21,404	2.55
P34	41,256	4.92
P51	46,116	5.50
P88	40,520	4.84
PA2	7773	0.93

## 4 Conclusions

In this study, a nanohybrid (NH34) using partially hydrolyzed polyacrylamide and nanosilica modified with 3-aminopropyltriethoxysilane was synthesized and characterized by using infrared spectroscopy and thermogravimetric analysis. For its application in EOR processes, the viscosifying power, mechanical stability, filterability, and emulsion behavior were evaluated along with adsorption in porous media, IPV, RF, and RRF. In laboratory tests, NH34 showed better mechanical and thermal degradation resistance than the EOR polymers P34, P51, P88, and PA2.

The water/oil separation process accelerates as the concentration of NH34 increases. This was attributed to the increase in the viscosity of the aqueous phase, causing greater resistance to its incorporation into the oleic phase. The water quality under producer well conditions and surface fluid treatment conditions (clarification) were affected (higher oil and grease content and turbidity) by the increased residual NH34 concentration. It was attributed to the higher viscosity of the aqueous phase, preventing the coalescence of the crude oil droplets. The dehydrated crude oil maintained the quality conditions (%BSW, viscosity, and drop size) of the control samples (brine). Hence, no adverse effects on the W/O emulsion are expected from the presence of residual NH34 concentration.

Adsorption in the porous media was PA2 > NH34 > P88 > P51 > P34, and IPV values were between 19 and 25%. For P34, PA2, and NH34, RF and RRF values increased with an increase in shear rate due to phenomena like mechanical entrapment and adsorption in the porous media, respectively. In contrast, the increased shear rate decreased RRF values for P88 polymer by removing polymer molecules retained in the porous media. Numerical simulation showed that polymers P51, P34, and P88 had better recovery factors than NH34 and PA2 due to their lower residual adsorption and IPV values.

In conclusion, the covalent grafting of nSiO_2_-APTES into the P34 structure increases its chemical and mechanical resistance. However, further experiments should be conducted to improve the nanohybrid′s performance and increase oil production with less chemical injection.

## Nomenclature

APTES: 3- aminopropyltriethoxysilane
C: polymer concentration on the effluents
CaCl_2_: calcium chloride
cEOR: chemical enhanced oil recovery
Co: initial polymer concentration
FTIR: Fourier-transform infrared spectroscopy
HPAM: partially hydrolyzed polyacrylamide
INJ: injection well
IPV: inaccessible pore volume
KCl: potassium chloride
MgCl_2_: magnesium chloride
NaCl: sodium chloride
NH34: nanohybrid
NP: nanoparticle
P1–P4: production wells 1–4
PV: pore volume
PVI: pore volume injected
Q: flow rate
PV: pore volume
PVI: pore volume injected
R: capillary radius
RF: resistance factor
RRF: residual resistance factor
TGA: thermogravimetric analysis
ΔP_w_: pressure drop during brine injection
ΔP_wp_: pressure drop during brine injection after polymer flooding
ΔP_p_: pressure drop during polymer or nanopolymer sol injection
**Greek letters**
γ̇: shear rate
